# Automated segmentation of the fractured vertebrae on CT and its applicability in a radiomics model to predict fracture malignancy

**DOI:** 10.1038/s41598-022-10807-7

**Published:** 2022-04-25

**Authors:** Taeyong Park, Min A Yoon, Young Chul Cho, Su Jung Ham, Yousun Ko, Sehee Kim, Heeryeol Jeong, Jeongjin Lee

**Affiliations:** 1 Department of Radiology and Research Institute of Radiology, University of Ulsan College of Medicine, Asan Medical Center, 88 Olympic-ro 43-gil, Songpa-gu, Seoul, 05505 Korea; 2 Biomedical Research Center, Asan Institute for Life Sciences, Asan Medical Center, 88 Olympic-ro 43-gil, Songpa-gu, Seoul, 05505 Korea; 3 Department of Clinical Epidemiology and Biostatistics, Asan Medical Center, 88 Olympic-ro 43-gil, Songpa-gu, Seoul, 05505 Korea; 4 School of Computer Science and Engineering, Soongsil University, 369 Sangdo-ro, Dongjak-gu, Seoul, 156-743 Korea

**Keywords:** Bone cancer, Metastasis, Musculoskeletal system, Trauma, Computational science

## Abstract

Although CT radiomics has shown promising results in the evaluation of vertebral fractures, the need for manual segmentation of fractured vertebrae limited the routine clinical implementation of radiomics. Therefore, automated segmentation of fractured vertebrae is needed for successful clinical use of radiomics. In this study, we aimed to develop and validate an automated algorithm for segmentation of fractured vertebral bodies on CT, and to evaluate the applicability of the algorithm in a radiomics prediction model to differentiate benign and malignant fractures. A convolutional neural network was trained to perform automated segmentation of fractured vertebral bodies using 341 vertebrae with benign or malignant fractures from 158 patients, and was validated on independent test sets (internal test, 86 vertebrae [59 patients]; external test, 102 vertebrae [59 patients]). Then, a radiomics model predicting fracture malignancy on CT was constructed, and the prediction performance was compared between automated and human expert segmentations. The algorithm achieved good agreement with human expert segmentation at testing (Dice similarity coefficient, 0.93–0.94; cross-sectional area error, 2.66–2.97%; average surface distance, 0.40–0.54 mm). The radiomics model demonstrated good performance in the training set (AUC, 0.93). In the test sets, automated and human expert segmentations showed comparable prediction performances (AUC, internal test, 0.80 vs 0.87, p = 0.044; external test, 0.83 vs 0.80, p = 0.37). In summary, we developed and validated an automated segmentation algorithm that showed comparable performance to human expert segmentation in a CT radiomics model to predict fracture malignancy, which may enable more practical clinical utilization of radiomics.

## Introduction

Radiomics is a multi-step process of converting medical images into meaningful and mineable data^1,2^. In the hand-crafted radiomics pipeline, the process includes segmentation, feature extraction, feature selection, and construction of diagnostic, prognostic or predictive models. Radiomics has shown promising results in oncologic imaging as a tool to reflect the tissue heterogeneity and its application to other medical fields, including spine imaging, has been growing^1,2^.

Segmentation is the most fundamental process of radiomics analysis, as subsequent feature extraction is based on segmented volumes and, consequently, affects the performance of the prediction model^2–5^. Reliable and reproducible segmentation is therefore essential for robust feature extraction and radiomics analysis. Image segmentation in radiomics can be performed manually, semi-automatically using methods such as region-growing or thresholding, or fully automatically using deep learning algorithms^1^. Although manual segmentation methods have been commonly used for the radiomics analysis of vertebrae^6–9^, manual delineation of the VOI is labor-intensive and time-consuming, especially for thin-slice CT of the spine yielding a large number of reconstructed images, making it prone to intra- and/or inter-observer variability^10^. Several automated approaches, including statistical shape models^11^, atlas-based methods^12^, active contours^13^, and intensity-based level-sets^14^, have been used for vertebral segmentation. With increasing application of machine learning in imaging processing, machine learning algorithms have also been used, mainly for detection of vertebrae^15,16^. More recently, deep learning has been more widely used for automated vertebral segmentation. A fully automated segmentation method using convolutional neural network (CNN) was shown to result in more reproducible and time efficient segmentation than manual segmentation^17^, and several studies have shown favorable results with Dice similarity coefficients (DSC) > 90% in the segmentation of intact, non-fractured vertebrae^18–21^. In more recent studies, deep learning algorithms were benchmarked on more diverse datasets including benign fractured vertebrae^22–24^. Lessmann et al.^23^ proposed a single stage vertebral segmentation method based on an iterative fully convolutional neural network and showed average DSC of 94.9%, and Payer et al.^24^ performed a three-step fully automatic approach combining SpationalConfig-Net and U-net for spine localization and segmentation and achieved overall DSC of 94%. However, literature on three-dimensional automated segmentation of metastatic vertebrae is limited. Gordon et al. used atlas-based method for segmentation of metastatic spine and achieved 87.67–96.22% concurrency^25^. More recently, Klein et al. used a three-dimensional U-net CNN to automatically segment metastatic trabecular bone on CT with DSC of 90.4%^26^; however, metastatic spine with malignant fractures were excluded from the cohort.

Differentiation of benign and malignant compression fracture is a frequently encountered problem in clinical practice. Accurate diagnosis is important with a considerable difference in management and prognosis. In particular, it is increasingly important but challenging to differentiate benign and malignant fractures in elderly population with both high prevalence of osteoporosis and high cancer incidence rates. Imaging modalities such as CT and MRI play an important role in determining the benignity or malignancy of vertebral fractures. The widespread availability, speed and affordability of CT have led to its frequent use in the evaluation of vertebral fractures. In several recent studies, CT radiomics has shown promising results in the evaluation of vertebral fractures^6,9^, including the ability to successfully differentiate malignant from acute benign compression fractures^6^. These findings suggest that CT radiomics may provide an alternative diagnostic approach to determine the etiology of vertebral fractures. In that study, however, the fractured vertebrae were segmented manually, limiting the routine clinical implementation of the proposed prediction model. We hypothesized that this limitation could be overcome by automated segmentation, potentially leading to more successful implementation of radiomics in clinical practice.

Therefore, in this study, we aimed to develop and validate an automated algorithm for segmentation of fractured vertebral bodies on CT. Additionally, to evaluate the applicability of automated algorithm for use in radiomics, the algorithm was compared with the human expert segmentation for the prediction performance of a radiomics model to differentiate between acute benign and malignant compression fractures.

## Methods

### Patients

This retrospective study was approved by the Institutional Review Board of the Asan Medical Center (approval no. 2019-0134), Institutional Review Board of the Seoul National University Bundang Hospital (no. B-2008/628-109) and Institutional Review Board of the Inha University Hospital (no. 2020-08-018), and the requirement to obtain informed patient consent was waived. All methods were performed in accordance with the relevant guidelines and regulations.

**Figure 1:**
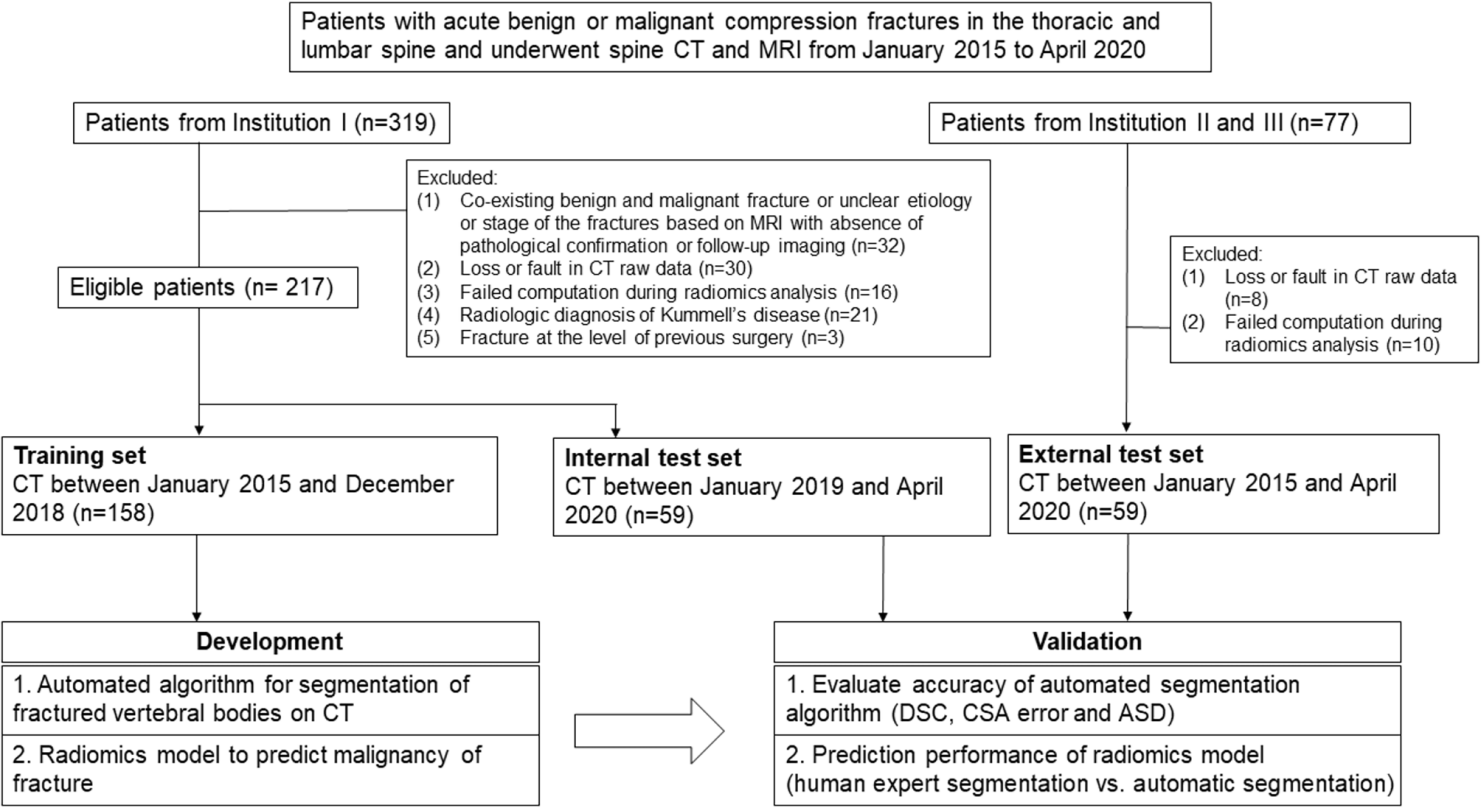
Flow diagram of the study.

This study included patients who (a) underwent spine CT for acute benign or malignant vertebral compression fractures in the thoracic and lumbar vertebrae between January 2015 and April 2020, and (b) underwent MRI within 6 weeks of CT examination. Acute benign compression fractures were defined as traumatic or osteoporotic fractures with abrupt onset of back pain of less than 6 weeks^27^, whereas malignant fractures were defined as fractures replaced or infiltrated by tumor tissue^28^. In addition, chronic fractures were defined as old, healed benign compression fractures without bone marrow edema on MRI. Patient exclusion criteria are shown in the flow diagram (Fig. 1).

An automated algorithm to segment fractured vertebral bodies was developed using a training set of consecutive patients who underwent CT scans between January 2015 and December 2018 at one tertiary referral center (Institution I: Asan Medical Center). The generalizability of our algorithm was tested on two independent test sets: an internal test set of consecutive patients who underwent CT scans between January 2019 and April 2020 at the same center (Institution I), and an external test set of randomly sampled patients from two other tertiary referral centers (Institutions II and III: Seoul National University Bundang Hospital and Inha University Hospital). It has been suggested that, in radiomics, the number of patients in the external test set be 25–40% of the number in the training set^29^. Therefore, the external test set consisted of about 50% of the number of patients in the training set, with consideration of possible further exclusion.

### Reference standard

The benignity or malignancy and the acuity or chronicity of fracture was determined by a musculoskeletal radiologist with 10 years of experience in spine imaging, based on MRI performed within 6 weeks of CT examination, and, if available, follow-up imaging or pathologic confirmation of tissue samples obtained surgically or on percutaneous biopsies.

Acute benign fractures were diagnosed when patients had (a) unequivocal MRI findings as described in previous literature^28,30,31^ and/or (b) healing of the fracture with fatty marrow restoration on follow-up imaging. Malignant fractures were diagnosed when patients had (a) unequivocal MRI findings shown in previous studies^28,30,31^, (b) disease progression on serial MRI, and/or (c) pathologic confirmation of malignancy.

### Ground truth segmentation

Ground truth segmentation was performed manually by two expert image analysts with 2–3 years of experience in medial image segmentation and who were blinded to the pathological results. For each vertebra with fracture, a three-dimensional VOI was drawn along the outer margins of the vertebral body and at the anterior margin of pedicles on axial CT images of 1 or 1.25 mm thickness. If CT showed chronic features in patients with acute benign fracture, these chronic features were also segmented, yielding 529 ground truth labels. Finally, all segmented images were re-evaluated and approved by a board-certified musculoskeletal radiologist. Segmentation was performed using in-house software (AsanJ), a plugin for the open source image processing program ImageJ (http://rsb.info.nih.gov/ij/).

### CT protocol

The details of the CT protocols are presented in Table 1.

**Table 1:** Details of CT protocols.

Scanner	Somatom Sensation 16, Somatom Definition Edge, Flash, Force, AS or AS+ (Siemens Healthineers)	LightSpeed VCT, Optima CT660 or Discovery CT750HD (GE Healthcare)
Tube voltage (kVp)	120	120
Time–current product	Care Dose 4D with quality reference mAs of 200	auto mA and Smart mA (minimum of 100 and maximum of 400 mA) with a noise index set to 21.0 HU
Detector collimation (mm)	0.6	1.25
Rotation time (s)	0.5	0.5
Pitch	1.0	0.97
Reconstruction	Axial plane at 1 mm slice thicknesses with 0.7 mm increments using a standard kernel (B30 filter)	Axial plane at 1.25 mm slice thicknesses with 0.8 mm increments using a standard kernel
Voxel size (mm)	0.293 × 0.293 × 1 (FOV 150 × 150) (most commonly used) (range, 0.287 × 0.287 × 1 [FOV, 147 × 147] − 0.324 × 0.324 × 1 [FOV, 166 × 166])	
Matrix	512 × 512	

### Development of automated algorithm for fractured vertebral body segmentation

**Figure 2:**
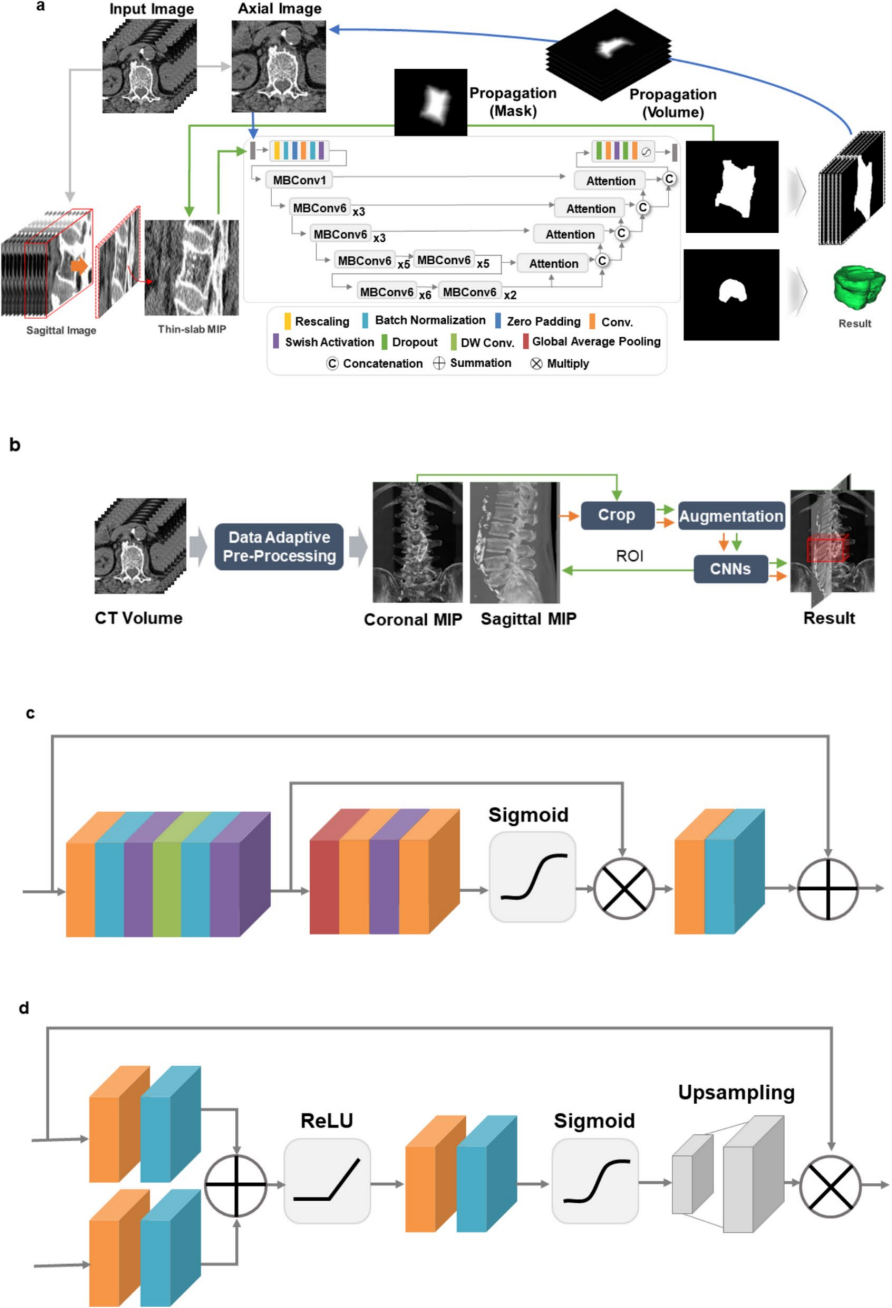
The proposed convolutional neural network (CNN) to segment fractured vertebral bodies on CT. (**a**) Overview of the development of the CNN and its detailed architecture. (**b**) Overall process of vertebral detection. (**c**) MBConV block used for the encoding path. (**d**) Attention block used for the decoding path.

An overview of the development of the CNN and its detailed architecture are presented in Fig. 2. The proposed fractured vertebral body segmentation method was composed of two steps: vertebral detection and segmentation.

#### Vertebral detection

Prior to training the model, pre-processing was performed to generate consistent maximum intensity projection (MIP) images from CT images^32^. In pre-processing, Otsu thresholding, region growing, morphological filtering and histogram equalization were sequentially performed. MIP images in the coronal plane were cropped to 416 × 416 pixels. Cropping included consideration of the scale and rotation transformation to be used as augmentation, together with the cutout^33^. The cutout was applied to reflect the lost region of vertebrae due to fracture or regions affected by nearby metals^34^.

YoloV3 is a one-stage detector that uses multi-scaled feature maps and predefined anchor boxes to rapidly and accurately predict localization and class of bounding boxes^35^. The baseline consisted of the YoloV3 framework^35^, followed by the modifications that included (a) application of dense connection and separable convolution to the yolo block, and (b) effective reduction of the scale layer through data augmentation and optimization of the anchor box and grid size for vertebrae. These enabled efficient improvements of accuracy and rapid ROI extraction. Each vertebral ROI extracted from the coronal MIP image was used as a limit to generate a sagittal MIP image. Vertebral ROIs were extracted from the sagittal MIP images in the same manner as in the coronal plane. Vertebral VOIs were generated using the minima and maxima for the x, y, and z coordinates of each ROI extracted from the two planes of MIP images.

#### Fractured vertebral body segmentation

Because severe bone destruction in some cases made it difficult to determine the total morphology of each vertebra, segmentation was first performed in the sagittal plane, followed by the axial plane. Thin-slab MIPs were generated from continuous slices, followed by propagation of reduced segmentation areas to adjacent areas to improve segmentation performance. Thin-slab MIPs compensated for the partial loss or broken regions by merging information from the n-th adjacent slice. Propagation maintained the topologic characteristics of the overall fractured vertebral body based on the linear characteristics of the adjacent regions on CT images. The results of segmentation in the sagittal plane were used to reconstruct images in the axial plane. At this time, the CNN segmentation prediction area was reduced using the distance map in order to solve the over-segmentation problem caused by thin-slab MIP generation.

#### Base architecture

Our network was based on U-Net framework^36^. The CNN consisted of encoding and decoding paths, and the performance was improved through EfficientNet^37^ in the encoding path and Attention U-Net^38^ in the decoding path. Application of the compound scaling method to the encoding path of the proposed CNN architecture reduced calculation costs and improved accuracy. In the decoding path, the attention block emphasized important features of the vertebrae, progressively suppressing the feature response to the background area. The numbers of parameters were effectively reduced in the encoding and decoding paths, improving the performance. A total of five resolution steps were used in all experiments.

#### Loss function

Binary Cross Entropy (BCE) and Dice Loss^39^ were performed to minimize background bias in vertebral segmentation results. In addition, propagation loss was used to compensate for partially lost or broken regions. The overall loss function can be defined as:

$${\mathcal{L}} = \alpha {\mathcal{L}}_{bce} + \beta {\mathcal{L}}_{dice} + \gamma {\mathcal{L}}_{prop}$$

$${\mathcal{L}}_{bce} = - \frac{1}{N}\mathop \sum \limits_{i}^{N} \left( {y_{{g_{i} }} \log \left( {x_{{p_{i} }} } \right) + \left( {1 - y_{{g_{i} }} } \right)\log \left( {1 - x_{{p_{i} }} } \right)} \right)$$

$${\mathcal{L}}_{dice} = 1 - \frac{{2\mathop \sum \nolimits_{i}^{N} x_{{g_{i} }} y_{{g_{i} }} }}{{\mathop \sum \nolimits_{i}^{N} x_{{g_{i} }} + \mathop \sum \nolimits_{i}^{N} y_{{g_{i} }} }}$$

$${\mathcal{L}}_{prop} = \frac{{\mathop \sum \nolimits_{i}^{N} \left( {p_{{g_{i} }} - x_{{g_{i} }} p_{{g_{i} }} } \right)}}{{\mathop \sum \nolimits_{i}^{N} p_{{g_{i} }} }}$$

where ${\mathcal{L}}_{bce}$, ${\mathcal{L}}_{dice}$ and ${\mathcal{L}}_{prop}$ represent the BCE, dice, and propagation loss function, respectively, and $\alpha , \,\beta ,\, \gamma$ are the balancing coefficients. *N* represents the number of pixels. $x_{p } \in [ {0,\;1} ]$ represents predicted probability, and $x_{g } \in [ {0,\;1} ]$, $y_{g } \in [ {0,\;1} ]$, and $p_{g } \in [ {0,\;1} ]$ represent the predicted, ground truth, and propagation labels, respectively.

#### Learning the network

For the training data, CT images of 512 × 512 size were cropped and resized based on VOI and axial and sagittal images of 288 × 288 size were used. Augmentation was performed by randomly combining affine transformation, crop, and cutout^33^. Xavier uniform initialization^40^ and an Adam optimizer were used for network weight initialization and optimization, respectively, with the learning rate set at 3e−4. We set the scheduler's patience to 30 and decreased the learning rate by multiplying by 0.1 every 10 epochs. The network was trained for 100 epochs using Intel^®^ Core™ i7-8700 3.20 GHz processor, 32 GB RAM memory, and TITAN RTX 24 GB (NVIDIA, Santa Clara, CA, USA).

### Quantitative evaluation of automatic segmentation

The accuracy of the algorithm was evaluated on the internal and external test sets by using the DSC, cross-sectional area (CSA) error, and average surface distance (ASD).

DSC, a measure of spatial overlap between automatic segmentation (A) and ground truth (B) on a pixel-by-pixel basis, was calculated as^41^:

$${\text{DSC }}( {{\text{A}},{\text{ B}}} ) = 2| {A \cap B} |/( {| A | + | B |} )$$

CSA error, a measure of the percent difference in area between automatic segmentation (A) and ground truth (B), was calculated as^32^:

$${\text{CSA error }}( \% ) = ( {[ {{\text{B}}_{{{\text{CSA}}}} {-}{\text{ A}}_{{{\text{CSA}}}} } ]/{\text{ B}}_{{{\text{CSA}}}} } ) \times 100$$

ASD, the average minimal distance between points on the surfaces of automatic segmentation (A) and the ground truth (B), was calculated as^41^:

$${\text{ASD }} = \frac{1}{{| {\text{A}} |}}\mathop \sum \limits_{{{\text{s}}_{{\text{Y}}} \in {\text{S}}_{{\text{Y}}} }} {\text{d}}( {{\text{s}}_{{\text{A}}} ,{\text{ S}}_{{\text{B}}} } )$$

### Statistical analysis

Categorical variables were compared using the Chi-square test or Fisher’s exact test, as appropriate, and continuous variables were compared using two-sample *t* test or the Kruskall–Wallis ANOVA test. All statistical analyses were performed using R statistical software, version 3.6.3 (R Foundation for Statistical Computing, Vienna, Austria), with p-values < 0.05 considered statistically significant.

### Evaluation of applicability of the algorithm in radiomics

To evaluate the applicability of the algorithm in radiomics, the prediction performance between the automated and human expert segmentations was compared in terms of a radiomics prediction model to differentiate acute benign and malignant compression fractures. For radiomics analysis of patients with multiple fractures, one vertebra was randomly selected using the RAND function of Microsoft Excel (Microsoft Corporation, Redmond, WA, USA). A total of 280 radiomics features were extracted from each vertebra (Supplementary Table S1). To standardize voxel spacing, the images were resampled to a voxel size of 0.29 × 0.29 × 0.70 mm, and quantified to a quantization range of mean ± 3 × SD and 64 bins. Radiomics features were extracted using in-house software (AsanFEx) implemented in MATLAB (MathWorks, Natick, MA, USA). The radiomics features used in this study followed the guidelines of the image biomarker standardization initiative (IBSI).

#### Construction of a radiomics prediction model

A radiomics model predicting malignancy of compression fracture was constructed from the training set.

First, features with zero variation across patients were removed, and each of the remaining features was normalized to have zero mean and unit standard deviation.

Second, to select robust features with respect to segmentation, 35 randomly chosen vertebral bodies (20 with acute benign and 15 with malignant fractures) were re-segmented by one of the three board-certified musculoskeletal radiologists, who were not involved in the creation of ground truth data. Intra-individual repeatability test using concordance correlation coefficient (CCC) is one of the recommended strategies to build more reproducible radiomics features and to reduce data dimensionality^17^. Therefore, highly stable features, defined as those with CCC > 0.90 between the ground truth labels and segmentation by three musculoskeletal radiologists, were retained for subsequent analysis.

Third, to prevent multicollinearity, univariable association with the fracture malignancy was examined for any highly-correlated (> 0.90) two features, and the feature with a larger p-value was excluded from subsequent analysis.

Then, fivefold cross-validation was performed using least absolute shrinkage and selection operator (LASSO) regression with penalty parameter tuning to select significant radiomics features with non-zero coefficients that can predict malignancy of vertebral fracture. Finally, a radiomics model was constructed from linear combinations of features weighted by LASSO coefficients.

#### Comparison of prediction performance

The diagnostic performance of the radiomics prediction model was compared between the automated and human expert segmentations on the internal and external test sets. The performance of the model was evaluated using the area under the receiver operating characteristics curve (AUC) and compared using the Delong method.

The diagnostic accuracy of the model for predicting malignant fracture at the optimal cutoff value derived by maximizing the Youden index (sensitivity + specificity − 1) was assessed. Accuracy, sensitivity, specificity, positive predictive value (PPV), and negative predictive value (NPV) were calculated, and the exact McMemar’s test was used to compare them.

## Results

### Patient characteristics

The algorithm was developed using a training set of 158 patients (mean ± standard deviation [SD] age, 66 ± 15 years; 92 women) with 341 vertebrae (one vertebra [n = 75 patients], two [n = 31], three [n = 29], four [n = 12], five [n = 5], six [n = 3], eight [n = 2], and ten [n = 1]). The algorithm was tested on a temporally independent internal test set of 59 patients (mean ± SD age, 63 ± 16 years; 31 women) with 86 vertebrae (one vertebra [n = 42 patients], two [n = 11], three [n = 3], four [n = 2], and five [n = 1]) and on a geographically independent external test set of 59 patients (mean ± SD age, 63 ± 16 years; 31 women) with 102 vertebrae (one vertebra [n = 35 patients], two [n = 15], three [n = 2], four [n = 4], and five [n = 3]) (Fig. 1).

The demographic and clinical characteristics of these three sets of patients are summarized in Table 2. The mean interval between CT and MRI was 5.9 days (range, 0–39 days).

**Table 2:** Baseline demographic and clinical characteristics of patients in the training and test sets. Data for age are means ± standard deviation.

	Training set (n = 158)			Test set (n = 118)					
	Benign (n = 84)	Malignant (n = 74)	*p*-value	Internal (n = 59)			External (n = 59)		
				Benign (n = 27)	Malignant (n = 32)	*p*-value	Benign (n = 29)	Malignant (n = 30)	*p*-value
Fractured bodies	188	153		37	49		56	46	
	(acute: chronic = 116:72)			(acute: chronic = 30:7)			(acute: chronic = 39:17)		
Vertebral levels (thoracic:lumbar)	Acute: 35:81, chronic: 31:41, malignant: 100:53			Acute: 9:21, chronic: 2:5, malignant: 28:21			Acute: 9:30, chronic: 6:11, malignant: 27:19		
Age (years)	72 ± 14	59 ± 12	< 0.001	67 ± 17	59 ± 14	0.08	66 ± 17	60 ± 15	0.15
Sex (men:women)	25:59	41:33	< 0.001	9:18	19:13	0.05	9:20	19:11	0.01
Origins of malignant fractures	Lung (n = 16), hepatobiliary (n = 13), multiple myeloma (n = 7), kidney (n = 6), colorectal (n = 6), breast (n = 5), stomach (n = 4), thyroid cancer (n = 3), neuroendocrine (n = 2), urothelial (n = 2), and others (n = 10)			Lung (n = 8), hepatobiliary (n = 6), breast (n = 5), prostate (n = 3), and others (n = 10)			Lung (n = 12), breast (n = 3), prostate (n = 3), hepatobiliary (n = 3), pancreas (n = 2), multiple myeloma (n = 2), and others (n = 5)		

### Performance of the automated algorithm for segmentation of fractured vertebrae

**Figure 3:**
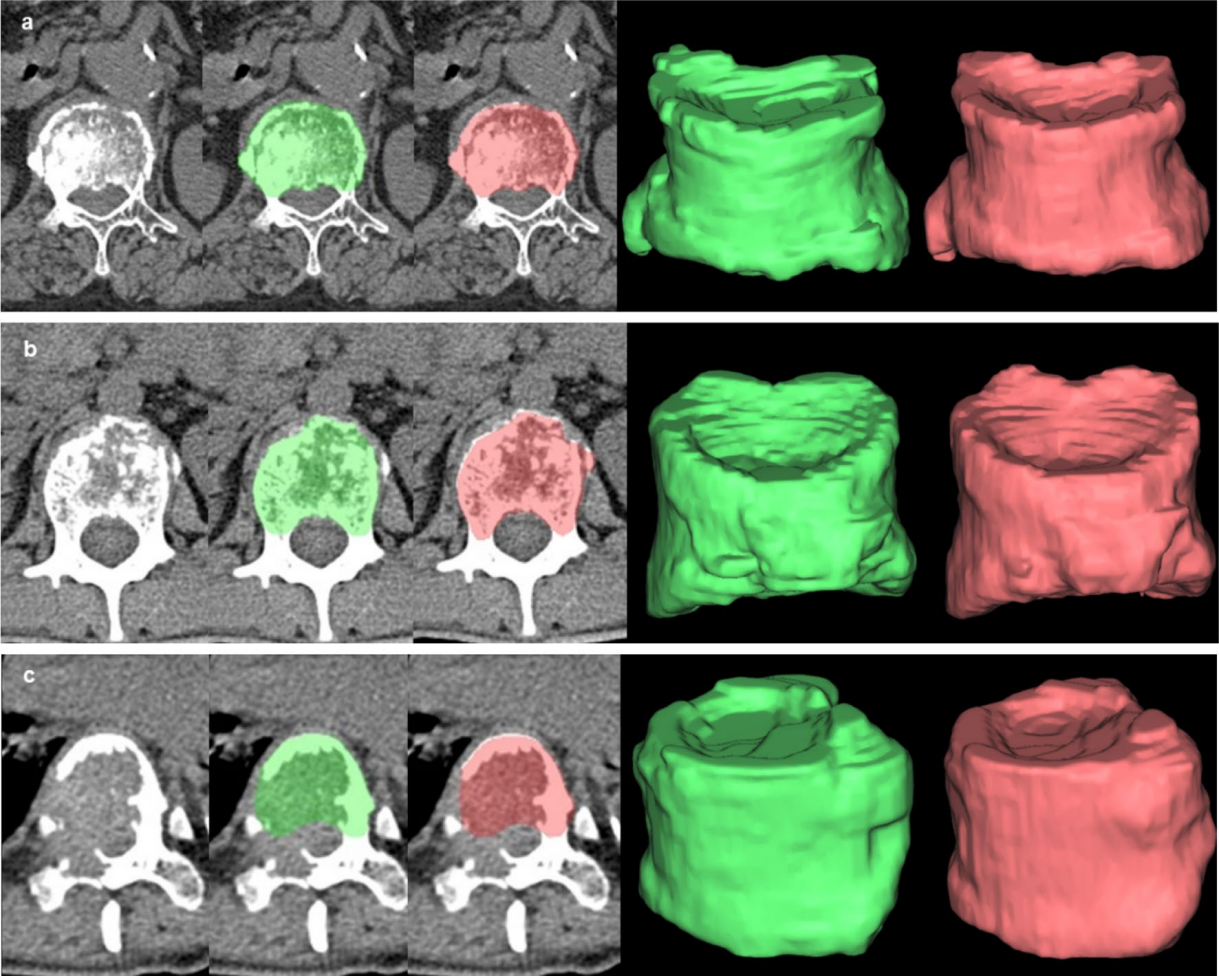
Representative images of automated segmentation of fractured vertebral bodies from the internal test set. (**a**) a 76-year-old woman with an acute benign fracture (voxel size, 0.287 × 0.287 × 1 mm), (**b**) a 19-year-old man with a malignant fracture from metastatic Ewing sarcoma/PNET (voxel size, 0.293 × 0.293 × 1 mm), and (**c**) a 68-year-old man with a malignant fracture from metastatic renal cell carcinoma (voxel size, 0.309 × 309 × 1 mm). When the osseous margin of the vertebral body could not be fully traced because of bone destruction, an imaginary line was drawn based on the contralateral normal appearing cortex or the most adjacent intact vertebral body as shown in (**c**). The green shaded area denotes segmentation by the human experts and the red shaded area denotes automated segmentation. The last two columns show three-dimensional volume meshes by the human experts (green) and the automated algorithm (red).

The accuracy of the deep-leaning based automated segmentation algorithm is summarized in Table 3. The algorithm achieved high agreement with the ground truth by human experts for segmentation of fractured vertebral bodies on the two independent test sets, with overall median DSCs of 0.94 and 0.93, CSA errors of 2.66% and 2.97%, and ASDs of 0.40 mm and 0.54 mm, on the internal test and the external test, respectively. Representative images of automated segmentation of fractured vertebral bodies are shown in Fig. 3. The median runtime for automated segmentation of a vertebral body with fracture 1.18 s (range, 0.87–1.51 s).

**Table 3:** Accuracy of automated segmentation algorithm for fractured vertebral body segmentation. All results are shown as median and interquartile ranges in brackets. DSC indicates dice similarity coefficient; CSA, cross-sectional area; ASD, average surface distance. ^a^p-value for comparison between chronic benign, acute benign and malignant fractures.

	Internal test set					External test set				
	Overall	Chronic Benign	Acute Benign	Malignant	*p*-value^a^	Overall	Chronic Benign	Acute Benign	Malignant	*p*-value^a^
	(n = 86)	(n = 7)	(n = 30)	(n = 49)		(n = 102)	(n = 17)	(n = 39)	(n = 46)	
DSC	0.94 [0.92, 0.95]	0.95 [0.94, 0.95]	0.94 [0.93, 0.95]	0.93 [0.90, 0.95]	0.02	0.93 [0.92, 0.95]	0.94 [0.92, 0.95]	0.94 [0.93, 0.95]	0.93 [0.88, 0.94]	< 0.001
CSA error (%)	2.66 [1.32, 4.43]	3.24 [0.15, 3.41]	3.14 [1.62, 4.23]	2.63 [1.19, 5.02]	0.40	2.97 [1.09, 4.96]	2.05 [0.64, 3.97]	2.51 [0.93, 4.01]	3.92 [1.91, 7.68]	0.01
ASD (mm)	0.40 [0.32, 0.55]	0.38 [0.32, 0.40]	0.35 [0.31, 0.39]	0.48 [0.38, 0.62]	< 0.001	0.54 [0.42, 0.72]	0.48 [0.38, 0.63]	0.47 [0.36, 0.55]	0.63 [0.48, 0.99]	< 0.001

Subgroup analysis showed that the algorithm achieved the highest performance for chronic benign fractures, followed by acute benign fractures and malignant fractures, with statistically significant differences, except for the CSA error of the internal test set.

### Evaluation of applicability in radiomics

#### Construction of radiomics prediction model

Of the 280 radiomics features, 38 zero variance features and 175 unstable features with CCC > 0.90 from multiple observer segmentation were excluded, leaving 67 features for the subsequent analysis. Excluding one of the highly correlated features further reduced these 67 features to 39 features. Finally, the LASSO regression model selected 12 features, and their non-zero coefficients were used to construct a radiomics model to predict the fracture malignancy (Table 4).

**Table 4:** List of 12 radiomics features used to develop a radiomics model to predict malignancy of fracture. LoG indicates Laplacian of Gaussian Filtered features.

Feature family	Feature name	LASSO coefficient (β)
	Intercept	− 0.176
Morphological features	Approximate volume	− 0.483
	Major axis length	− 0.544
Local intensity features	Global intensity peak	0.837
Intensity-based statistical features	Minimum gray level	0.745
Intensity histogram features	Intensity histogram mean	0.730
	Intensity histogram robust mean absolute deviation	0.104
Gray level co-occurrence matrix	Joint entropy	− 0.088
Gray level size zone matrix	Small zone low gray level emphasis	− 0.994
Neighboring gray level dependence matrix	High dependence emphasis	0.329
LoG local intensity features	Local intensity peak	− 0.444
LoG intensity-based statistical features	75th percentile	− 0.105
LoG filtered intensity histogram features	Maximum histogram gradient	− 0.051

#### Model performance

The radiomics model showed good discriminatory performance in the training set (AUC, 0.93 [95% CI, 0.90–0.97]). Using the cutoff threshold of 0.328, the model showed accuracy of 85% (134/158), sensitivity of 93% (69/74), specificity of 77% (65/84), PPV of 78% (69/88), and NPV of 93% (65/70).

#### Comparison of automated and human expert segmentations

The diagnostic performances of the radiomics model in the test sets are shown in Table 5. Both the automated segmentation and the human expert segmentation yielded good AUCs of 0.80–0.87 in the test sets. In the internal test set, human expert segmentation showed slightly higher AUC than the automated segmentation, although the difference was statistically significant (AUC, 0.87 [95% CI, 0.78–0.96] vs. 0.80 [95% CI, 0.69–0.91]; p = 0.044). In the external test set, human expert segmentation and automated segmentation showed comparable performances (AUC, 0.80 [95% CI, 0.69–0.92] vs. 0.83 [95% CI, 0.72–0.94]; p = 0.37).

**Table 5:** Diagnostic performance of the radiomics prediction model. Numbers in brackets indicate 95% confidence interval.

	Internal test set (n = 59)			External test set. (n = 59)		
	Human expert segmentation	Automated segmentation	*p*-value	Human expert segmentation	Automated segmentation	*p*-value
AUC	0.87 [0.78, 0.96]	0.80 [0.69, 0.91]	0.044	0.80 [0.69, 0.92]	0.83 [0.72, 0.94]	0.37
Accuracy (%)	78 (46/59) [67, 89]	71 (42/59) [60, 83]	0.22	76 (45/59) [65, 87]	76 (45/59) [65, 87]	> 0.999
Sensitivity (%)	78 (25/32) [64, 92]	72 (23/32) [56, 88]	0.63	77 (23/30) [62, 92]	80 (24/30) [66, 94]	> 0.999
Specificity (%)	78 (21/27) [62, 94]	70 (19/27) [53, 88]	0.50	76 (22/29) [60, 91]	72 (21/29) [56, 89]	> 0.999
Positive predictive value (%)	81 (25/31) [67, 95]	74 (23/31) [59, 90]	N/A	77 (23/30) [62, 92]	75 (24/32) [60, 90]	N/A
Negative predictive value (%)	75 (21/28) [59, 91]	68 (19/28) [51, 85]	N/A	76 (22/29) [60, 91]	78 (21/27) [62, 94]	N/A

At the optimal cutoff thresholds, the classification performances of the automated and human expert segmentations were found to be comparable in accuracy (71–76% vs 76–78%), sensitivity (72–80% vs 77–78%), and specificity (70–72% vs 76–78%) (all p > 0.05 for comparison between segmentation methods).

## Discussion

In this study, we developed and validated an automated algorithm for segmentation of fractured vertebral bodies on CT. The algorithm achieved high agreement with the human expert segmentation on two independent test sets. In addition, the automated and the human expert segmentation methods were compared for the prediction performance of a radiomics model to differentiate acute benign and malignant compression fractures, and the two segmentation methods showed comparable discrimination performance and accuracy, indicating the applicability of the proposed algorithm for use in radiomics.

Automated segmentation is considered superior to manual or semi-automated segmentations for radiomics, with optimal reproducibility and time efficiency^17^. Several deep learning algorithms were found to be highly accurate in segmentation of intact and non-fractured vertebrae on CT with DSCs > 0.90^18–21^. However, segmentation of fractured vertebrae on CT is more challenging due to variations and complexity in morphology, low contrast in soft tissue, and more variable fields-of-view among patients. To date, few studies have attempted to segment fractured vertebrae on CT^41,42^. In one study, an algorithm trained on ten normal individuals achieved DSCs of 0.88–0.92 in five patients with a total of 16 osteoporotic compression fractures^42^. More recently, an algorithm trained on patients with benign fractures in two CT datasets showed a DSC of 0.93 and an ASD of 0.41 mm^41^. These studies, however, did not provide specific results on the segmentation of fractured vertebrae alone or detailed information on acuity or chronicity of fractures. To our knowledge, automated algorithm for segmentation of fractured vertebral bodies of various etiologies and stages, including malignant fractures, using relatively large training sets has not previously been well established in the literature. Moreover, the accuracy of our algorithm in segmentation of fractured bodies reached that previously reported for segmentation of normal, non-fractured vertebral bodies (DSC, 0.94)^42^.

Automated vertebral segmentation is needed for many purposes, including diagnosis and treatment planning. However, as Rizzo et al. mentioned, there is no universal segmentation algorithm for all applications and purposes^43^. We sought to develop an algorithm for subsequent use in radiomics analysis to differentiate acute benign and malignant compression fractures. While several previous works on automated segmentation only evaluated the reproducibility of radiomics features (i.e., correlations between automated and manual segmentation) as one measure of segmentation performance^5,44^, the extent of feature reproducibility may not directly translate into the performance of a radiomics model. Therefore, automated segmentation was compared with human expert segmentation in the performance of a radiomics model, which was constructed with features robust against segmentation variability, and the algorithm and the human experts showed comparable performances. These results suggest the applicability of the automated algorithm for use in a radiomics prediction model.

This study had several limitations. First, its retrospective design suggests a possibility of selection and referral bias. The study included only those patients who were evaluated by both CT and MRI within a short period of time. Patients who could be diagnosed by either modality alone often did not undergo further imaging evaluation and were therefore not included in the study population. Moreover, as all the patients enrolled in this study were from tertiary referral centers, the prevalence of malignant fractures was high. Second, although a previous study showed that the diagnostic performance of CT-based radiomics model for predicting fracture malignancy improved by integrating clinical parameters such as patient age and history of malignancy with radiomics features^6^, we developed the model using only the radiomics features, as the purpose of this study was to evaluate the applicability of the automated segmentation algorithm for use in radiomics. We believe that our radiomics model’s diagnostic accuracy measures can be improved by incorporating clinical parameters with radiomics features in the prediction model. Furthermore, in recent studies, machine learning algorithms have been applied in both feature selection and classification steps, and deep learning algorithms have been used for fully automated feature extraction and modeling steps without the need for further human intervention^45^. We believe that more automated approach using deep learning can be used for radiomics analysis to differentiate benign and malignant fractures. Finally, patients with failed computation during radiomics analysis were excluded from the cohort. One possible reason for computation failure would be a technical limitation of the software used in this study. As there were variations in image acquisition techniques and reconstruction parameters between institutions, some of the CTs from the institution II and III had a large axial fields-of-view and/or a large scan coverage in the cranio-caudal axis (whole spine CT scan). We experienced errors while uploading or standardizing voxel spacing of large thin-slice CT datasets. Moreover, for some features, there were errors during the feature extraction step. One previous study that examined the properties of failed radiomics feature extraction suggested that several factors such as the size of the ROI and high skewness of intensities may result in computational errors^46^. We suspect that certain physical properties of the fractured vertebrae and size of the ROIs could have caused feature extraction errors.

In conclusion, we developed and validated an automated algorithm for segmentation of fractured vertebral bodies on CT. The automated algorithm showed comparable performance to the human expert segmentation in a CT radiomics model to predict fracture malignancy, which may enable more practical clinical utilization of radiomics.

## Supplementary Information

Supplementary Table S1.

## Data Availability

The datasets generated during and/or analysed during the current study are available from the corresponding author on reasonable request.
