# Publisher Correction: Automated segmentation of the fractured vertebrae on CT and its applicability in a radiomics model to predict fracture malignancy

**DOI:** 10.1038/s41598-022-11784-7

**Published:** 2022-05-03

**Authors:** Taeyong Park, Min A Yoon, Young Chul Cho, Su Jung Ham, Yousun Ko, Sehee Kim, Heeryeol Jeong, Jeongjin Lee

**Affiliations:** 1 Department of Radiology and Research Institute of Radiology, University of Ulsan College of Medicine, Asan Medical Center, 88 Olympic‑ro 43‑gil, Songpa‑gu, Seoul, 05505 Korea; 2 Biomedical Research Center, Asan Institute for Life Sciences, Asan Medical Center, 88 Olympic‑ro 43‑gil, Songpa‑gu, Seoul, 05505 Korea; 3 Department of Clinical Epidemiology and Biostatistics, Asan Medical Center, 88 Olympic‑ro 43‑gil, Songpa‑gu, Seoul, 05505 Korea; 4 School of Computer Science and Engineering, Soongsil University, 369 Sangdo‑ro, Dongjak‑gu, Seoul, 156‑743 Korea

Correction to: *Scientific Reports* 10.1038/s41598-022-10807-7, published online 25 April 2022

The original version of this Article contained an error in the spelling of the author Min A Yoon which was incorrectly given as Min A. Yoon.

The original Article has been corrected.

