# Supplementary material for: Automated segmentation of the fractured vertebrae on CT and its applicability in a radiomics model to predict fracture malignancy
Source: Sci Rep. 2022 Apr 25;12:6735. doi: 10.1038/s41598-022-10807-7 (PMC9038736; doi:10.1038/s41598-022-10807-7)
Supplement: Supplementary file 1 — Supplementary Table S1. [file 41598_2022_10807_MOESM1_ESM.pdf]

**Supplementary Table S1.** List of the 280 radiomics features extracted from each vertebra.

| Family name | Feature name |
|---|---|
| Morphological features (Original image) | Volume, Approximate volume, Surface Area, Surface to volume ratio, Compactness 1, Compactness 2, Spherical disproportion, Sphericity, Asphericity, Major axis length, Minor axis length, Least axis length, Elongation, Flatness |
| Local intensity features (Original and LoG filtered) | Local Intensity peak, Global Intensity peak |
| Intensity-based statistical features (Original and LoG filtered) | Mean, Variance, Skewness, Kurtosis, Median<br>Minimum gray level, Maximum gray level, Range, 10th percentile, 25th percentile, 75th percentile, 90th percentile, Interquartile range, Mean absolute deviation, Median absolute deviation, Robust mean absolute deviation, Coefficient of variation, Quartile coefficient of dispersion, Energy, Root mean square |
| Intensity histogram features (Original and LoG filtered) | Intensity histogram mean, Intensity histogram variance, Intensity histogram skewness, Intensity histogram kurtosis, Intensity histogram median, Intensity histogram Minimum gray level, Intensity histogram mean absolute deviation, Intensity histogram maximum gray level, Intensity histogram range, Intensity histogram 10th percentile, Intensity histogram 25th percentile, Intensity histogram 75th percentile, Intensity histogram 90th percentile, Intensity histogram Interquartile range, Intensity histogram median absolute deviation, Intensity histogram robust mean absolute deviation, Intensity histogram coefficient of variation, Intensity histogram quartile coefficient of dispersion, Intensity histogram mode, Intensity histogram entropy, Intensity histogram uniformity, Maximum histogram gradient, Maximum histogram gradient gray level, Minimum histogram gradient gray level |
| Gray level co-occurrence-based features (Original and LoG filtered) | Angular second moment (energy, uniformity), Autocorrelation, Cluster prominence, Cluster shade, Cluster tendency, Contrast, Correlation, Difference average, Difference entropy, Difference variance, Dissimilarity (Difference average), Joint entropy, Harralick Correlation, Inverse difference (Homogeneity), First measure of information correlation, Second measure of information correlation, Inverse difference moment (Homogeneity), Normalized inverse difference moment, Normalized inverse difference, Inverse variance, Joint |

| | |
|---|---|
| | maximum, Joint average, Sum average, Sum entropy, Sum variance, Sum of squares (Joint variance) |
| Gray level run length-based features (Original and LoG filtered) | Gray level non-uniformity, High gray level run emphasis, Long runs emphasis, Long run high gray level emphasis, Long run low gray level emphasis, Low gray level run emphasis, Normalized run length non-uniformity, Run length non-uniformity, Run percentage, Short runs emphasis, Short run high gray level emphasis, Short run low gray level emphasis |
| Gray level size zone-based features (Original and LoG filtered) | Small zone emphasis, Large zone emphasis, Low gray level zone emphasis, high gray level zone emphasis, Small zone Low gray level emphasis, Small zone High gray level emphasis, Large zone low gray level emphasis, Large zone high gray level emphasis, Gray level non-uniformity, Normalized gray level non-uniformity, Zone size non-uniformity, Normalized zone size non-uniformity, Zone percentage, Gray level variance, Zone size variance, Zone size entropy |
| Gray level distance zone-based features (Original and LoG filtered) | Small distance emphasis, Large distance emphasis, Low gray level zone emphasis, high gray level zone emphasis, Small distance Low gray level emphasis, Small distance High gray level emphasis, Large distance low gray level emphasis, Large distance high gray level emphasis, Gray level non-uniformity, Normalized gray level non-uniformity, Zone distance non-uniformity, Zone distance non-uniformity normalized, Zone percentage, Gray level variance, Zone distance variance, Zone distance entropy |
| Neighboring gray level dependence-based features (Original and LoG filtered) | Low dependence emphasis, High dependence emphasis, Low gray level count emphasis, High gray level count emphasis, Low dependence low gray level emphasis, Low dependence high gray level emphasis, High dependence low gray level emphasis, High dependence high gray level emphasis, Gray level non-uniformity, Normalized gray level non-uniformity, Dependence count non-uniformity, Dependence count non-uniformity normalized, Dependence count percentage, Gray level variance, Dependence count variance, Dependence count entropy, Dependence count energy |

*LoG*, Laplacian of Gaussian filtered features.
